# Cell Cycle Arrest is a Conserved Function of Norovirus VPg Proteins

**DOI:** 10.3390/v11030217

**Published:** 2019-03-04

**Authors:** Alice McSweeney, Colin Davies, Vernon K. Ward

**Affiliations:** Department of Microbiology & Immunology, School of Biomedical Sciences, University of Otago, Dunedin 9054, New Zealand; alice.mcsweeney@postgrad.otago.ac.nz (A.M.); ctrd2@cam.ac.uk (C.D.)

**Keywords:** cell cycle, norovirus, *Caliciviridae*, VPg, G0/G1, MNV

## Abstract

Murine norovirus (MNV) viral protein genome-linked (VPg) manipulates the cell cycle to induce a G0/G1 arrest and gain a beneficial replication environment. All viruses of the norovirus genus encode a VPg protein; however, it is unknown if the G0/G1 arrest induced by MNV VPg is conserved in other members of the genus. RNA transcripts encoding a representative viral VPg from five norovirus genogroups were transfected into RAW-Blue murine macrophages, and the percentage of cells in each phase of the cell cycle was determined. A G0/G1 cell cycle arrest was observed for all norovirus VPg proteins tested, and in the wider *Caliciviridae* family the arrest was also conserved in rabbit hemorrhagic disease virus (RHDV) VPg and human sapovirus (HuSV) VPg. Truncation of MNV VPg shows that the first 62 amino acids are sufficient for a cell cycle arrest, and alignment of VPg sequences revealed a conserved motif in the N-terminal region of VPg. Analysis of VPg constructs with single N-terminal region point mutations, or exchange of N-terminal regions between VPg proteins, confirmed the importance of the N-terminal region for cell cycle arrest. These results provide evidence that G0/G1 cell cycle arrest is a conserved function of norovirus VPg proteins that involves the N-terminal region of these proteins.

## 1. Introduction

Noroviruses are a genus of the *Caliciviridae* family, which also includes the *Nebovirus*, *Lagovirus*, *Vesivirus* and *Sapovirus* genera [1]. The norovirus genus is further divided into at least five genogroups (GI–V), infecting a diverse range of host organisms [1,2]. Globally, human noroviruses (HuNV) are a major cause of viral gastroenteritis, affecting people of all age groups [3]. Of these, viruses from GII genotype 4 (GII.4) are responsible for the majority of infections [4,5,6].

Despite advances in the development of in vitro cell culture systems for HuNV, including B cells and stem cell-derived human enteroids, direct study of the virus remains challenging [7,8,9,10]. Consequently, murine norovirus (MNV) is often used as a model virus, as it retains a similar genetic layout to HuNV and exhibits robust replication in cell culture systems [11,12,13]. The norovirus genome is organized into three open reading frames (ORF). ORF1 encodes a large polyprotein, which is subsequently cleaved by the viral protease into the non-structural proteins NS1-2, NS3, NS4, NS5 (VPg), NS6, and NS7 [13]. ORF2 and ORF3 encode the major and minor capsid proteins, respectively. MNV also has an additional fourth ORF encoding a virulence factor (VF1) thought to be important in evading the host immune response [14,15].

Recently, it was shown that infection of a macrophage cell line with MNV results in a G0/G1 cell cycle arrest, and that expression of MNV viral protein genome-linked (VPg) alone is sufficient to induce the arrest [16,17]. MNV VPg is a multi-functional protein required for several important functions within the cell, including genome replication and viral protein translation. A conserved tyrosine residue at position 26 (Y26) of MNV VPg is thought to allow attachment of VPg to the 5′ viral RNA, and facilitate the function of VPg as a protein primer for viral RNA replication [18,19]. Substitution of Y26 with an alanine (Y26A) prevents the interaction of MNV VPg with viral RNA [18,20]. In the context of the cell cycle, a Y26A mutation has no effect on G0/G1 accumulation, suggesting that the cell cycle arrest does not require attachment of MNV VPg to the viral RNA [16]. A second, well-characterized function of MNV VPg is to recruit host eukaryotic initiation factors (eIFs) for preferential translation of the viral genome during infection [21,22]. The C-terminus of MNV VPg contains an ~20 amino acid motif, which directly interacts with the HEAT-1 domain of eIF4G [23]. Mutation of phenylalanine 123 (F123)within this motif substantially reduces binding to eIF4G; however, the same mutation has no effect on the cell cycle arrest induced by MNV VPg [16,24]. Taken together, this suggests that the cell cycle arrest is independent of two of the well-characterized functions of MNV VPg. 

Although all caliciviruses encode a VPg protein, it is unknown if the ability to manipulate the cell cycle is conserved. In this study, we expressed VPg proteins representing each of the norovirus genogroups and other calicivirus genera, and screened for the ability of each to cause a G0/G1 cell cycle arrest. We show that cell cycle manipulation by VPg is conserved within the norovirus genogroups, and selected VPg proteins of other genera of the calicivirus family. The ability of MNV VPg to manipulate the cell cycle was found to be associated with the N-terminal region of the protein—in particular, the first 10 amino acids.

## 2. Materials and Methods

### 2.1. Cell Culture

RAW-Blue murine macrophages (InvivoGen, San Diego, CA, United States), a derivative of RAW 264.7 cells, were cultured in Dulbecco’s modified Eagle’s medium (DMEM) supplemented with 10% (*v*/*v*) fetal bovine serum. Cells were passaged when they reached 70–80% confluency, and were maintained at 37 °C with 5% CO_2_.

### 2.2. Plasmid Constructs

Synthetic VPg plasmid constructs were designed with all of the requirements for in vitro RNA generation and synthesis (Table 1). A T7 promoter and a methionine were included at the 5′ end, and at the 3′ a stop codon followed by a restriction enzyme site for template linearization was included. Constructs were produced by Genscript (Genscript, Piscataway, NJ, USA) and cloned into a pUC57-simple vector. The plasmids were then transformed into XL1 Blue MRF’ *Escherichia coli* cells, and the plasmid DNA amplified by midi-prep (Invitrogen, Carlsbad, CA, United States).

Synthetic MNV VPg point mutation constructs were designed as above, but with single amino acid substitutions to alanine at lysine 5 or 7 (K5A or K7A), glycine 9 (G9A), and arginine 10 (R10A). Synthetic truncated MNV VPg constructs were designed to remove the indicated amino acids (Figure 3). Chimeric VPg constructs of MNV VPg with feline calicivirus (FCV) VPg or Newbury 1 VPg were designed around a conserved leucine residue (L21 of MNV VPg) (Figure 4). A C-terminal Strep-tag II epitope tag was incorporated for detection. All DNA constructs were sequenced to confirm correct identity prior to the preparation of RNA transcripts.

### 2.3. Preparation of RNA Transcripts

Plasmids were linearized at the 3′ end of the viral VPg gene using the appropriate restriction enzyme (Table 1). Messenger RNA transcripts were generated using the mMessage mMachine T7 ultra transcription kit (Ambion; ThermoFisher Scientific, Waltham, MA, USA) and purified using the MEGAclear transcription clean-up kit (Ambion), according to manufacturer’s instructions.

### 2.4. Transfection of RAW-Blue Cells

RAW-Blue cells were electroporated using the Neon transfection system, according to manufacturer’s instructions (ThermoFisher Scientific). Briefly, 1 × 10^6^ cells were mixed with 4–5 μg of in vitro transcript RNA, and electroporated with 1 pulse at 1730 V and 20 mA. Following transfection, cells were seeded into 2 mL of pre-warmed medium in a six-well plate, and incubated for the indicated time.

### 2.5. Confirmation of VPg Protein Expression

To confirm expression of VPg proteins, transfected cells were harvested and washed in Dulbecco’s phosphate buffered saline (dPBS), lysed, and then separated by SDS-PAGE electrophoresis. A broad-spectrum VPg antibody was not available to confirm expression of norovirus genogroup and calicivirus VPg proteins, and therefore SDS-PAGE gel samples were sent for mass spectrometry analysis (Centre for Protein Research, University of Otago, New Zealand). VPg constructs that could not be detected by mass spectrometry were redesigned to incorporate a 3′ Strep-tag II for western blot analysis (Table 1). MNV VPg was also available with a 3′ Strep-tag II. Cells transfected with tagged constructs were lysed, and the cell lysate clarified using MagStrep “type 3” XT beads (2-4090-002, IBA Lifesciences) and protein bound to those beads was analysed by western blot. Expression of Strep-tag constructs, MNV VPg, and derivates of MNV VPg were detected using the appropriate primary and secondary antibodies; Strep-tag II (34850; Qiagen, Hilden, Germany), MNV-1 VPg [25], actin (I-19) (sc1616; Santa Cruz; Santa Cruz, CA, United States), DyLight 800 donkey anti-rabbit IgG (SA5-10040; Thermo Scientific), and DyLight 680 donkey anti-mouse IgG (SA5-10090; Thermo Scientific). Images were viewed on the Odyssey Fc imaging system (LI-COR).

### 2.6. Cell Cycle Analysis

Following transfection, the percentage of cells in each phase of the cell cycle was measured by flow cytometry. Cells were harvested, washed in dPBS, and fixed overnight in 3 mL of cold 70% ethanol. Subsequently, the cells were washed and resuspended in dPBS. The DNA was stained with 50 μg/mL propidium iodide (P4170; Sigma) and the RNA degraded with 0.1 mg/mL RNase A (R4875; Sigma) for 45 min at 37 °C. Stained cells were analysed using fluorescence-activated cell sorting (FACS), and the cell cycle phases were assigned using MODfit LT 3.0 software (Verity Software House; Topsham, ME, United States).

### 2.7. Statistical Analysis

Data is presented as means and standard deviations. Results were analysed by a one-way ANOVA with Dunnett’s post-test, and *p* values of ≤0.05 were considered statistically significant.

### 2.8. Alignments

Alignments of VPg amino acid sequences were performed using Clustal omega software on the default settings and manually adjusted [26].

## 3. Results

**Figure 1:**
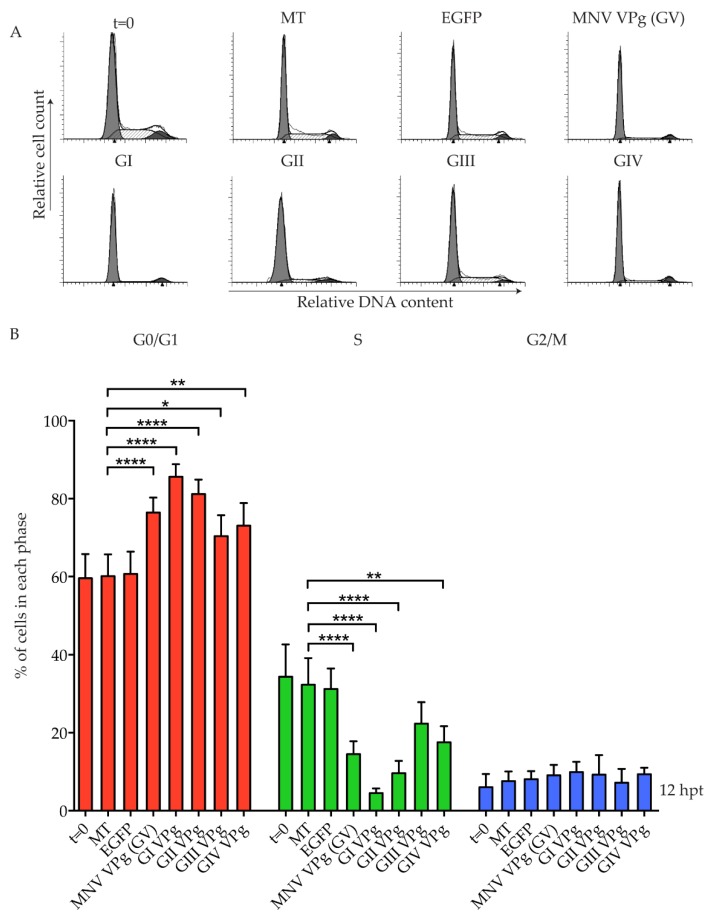
Conservation of a G0/G1 phase cell cycle arrest among norovirus genogroups. RAW-Blue cells were transfected with 4–5 µg of in vitro transcript RNA corresponding to enhanced green fluorescent protein (EGFP), murine norovirus (MNV) VPg, as well as genogroup (G) GI VPg, GII VPg, GIII VPg, or GIV VPg. Mock transfected (MT) cells were seeded at the time of transfection as a negative control. At 12 hours post-transfection (hpt), cells were harvested for fluorescence-activated cell sorting (FACS) analysis of the cell cycle. (**A**) Representative FACS histograms from one of three experiments. (**B**) The histograms were analysed using MODfit LT 3.0, and the percentage of cells in each phase of the cell cycle are shown. The results present the mean and SD from three independent experiments. Statistical significance was determined for comparisons between transfected cells and mock transfected cells, using a one-way ANOVA with Dunnett’s post-test. * *p* ≤ 0.05, ** *p* ≤ 0.01 and **** *p* ≤ 0.0001.

All viruses of the *Caliciviridae* family encode a VPg protein, but it is unknown if the cell cycle manipulation shown for MNV VPg is conserved. To determine if VPg proteins from other noroviruses are able to induce a G0/G1 cell cycle arrest, a single VPg was selected from each of the five norovirus genogroups (GI–V) and analysed for its effect on the cell cycle (Figure 1). VPg RNA was transfected into asynchronous RAW-Blue murine macrophages. Transfection of RNA transcripts encoding enhanced green fluorescent protein (EGFP) show a transfection efficiency of over 90%, as determined by FACS analysis [25]. As a broad-spectrum VPg antibody was not available, expression of VPg from genogroups GIII, GIV, and GV was confirmed by mass spectrometry analysis (Table S1). GI VPg and GII VPg could not be detected by mass spectrometry, and were subsequently epitope labelled with a Strep-tag II and purified on MagStrep beads to confirm expression by western blot (Figure S1). Two protein bands of similar molecular weights were observed for GI VPg (Figure S1). Analysis of the cell cycle by flow cytometry showed that at 12 hours post-transfection (hpt), all norovirus VPg proteins caused an increase in G0/G1 phase cells and a decrease in S phase cells (Figure 1B). In particular, VPg from GI and GII human noroviruses strongly increased the percentage of cells in G0/G1 to 85% and 81%, respectively, compared to mock transfected cells at 60%. GIII VPg from the Jena virus induced a modest though significant G0/G1 population increase, and a corresponding decrease in the S phase population was observed, although this was not significant (Figure 1B).

**Figure 2:**
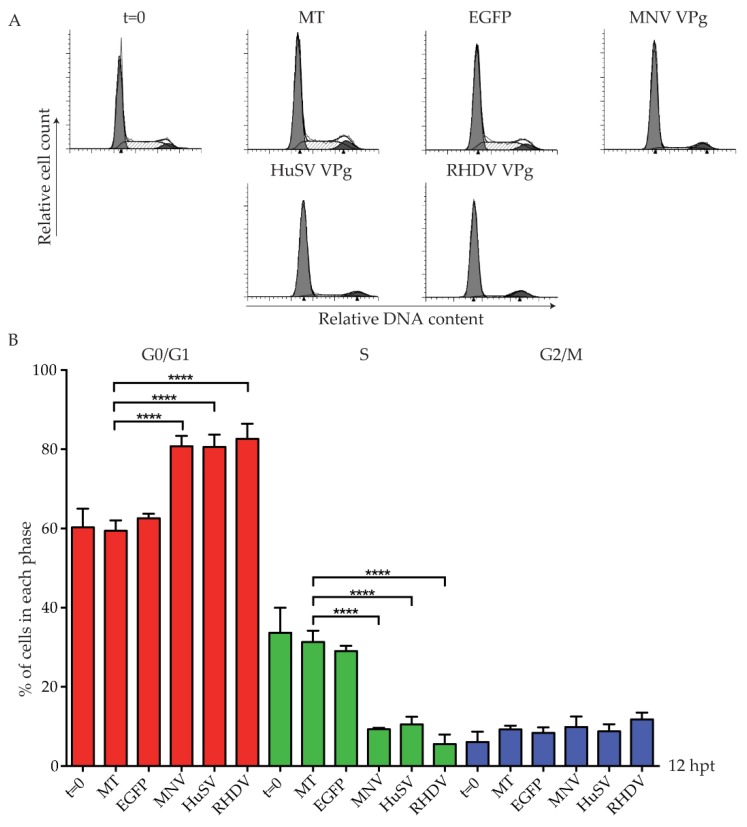
Human sapovirus (HuSV) VPg and rabbit hemorrhagic disease virus (RHDV) VPg induce a G0/G1 phase cell cycle arrest. RAW-Blue cells were transfected with 4–5 µg of in vitro transcript RNA corresponding to EGFP, MNV VPg, HuSV VPg, RHDV VPg, feline calicivirus (FCV) VPg, or Newbury1 VPg. Mock transfected (MT) cells were seeded at the time of transfection as a negative control. At 12 hpt, cells were harvested for FACS analysis of the cell cycle. (**A**) Representative FACS histograms from one of three experiments. (**B**) The histograms were analysed using MODfit LT 3.0, and the percentage of cells in each phase of the cell cycle are shown. The results present the mean and SD from three independent experiments. Statistical significance was determined for comparisons between transfected cells and mock transfected cells using a one-way ANOVA with Dunnett’s post-test. **** *p* ≤ 0.0001.

Previous work has demonstrated that VPg has conserved functions within the *Caliciviridae* family [27]. We were interested to investigate if the cell cycle arrest observed for members of the norovirus genus is conserved within other caliciviruses. A representative viral VPg from each of the five genera was tested, and the effect on the cell cycle analysed (Figure 2). Expression of human sapovirus (HuSV) VPg and rabbit hemorrhagic disease virus (RHDV) VPg was detected by mass spectrometry, and these proteins increased the G0/G1 population to 80% and 82%, respectively (Table S1; Figure 2B). Expression of Newbury 1 VPg and FCV VPg could not be confirmed by mass spectrometry, and Strep-tagged versions were not detected by western blot analysis (Figure S1).

**Figure 3:**
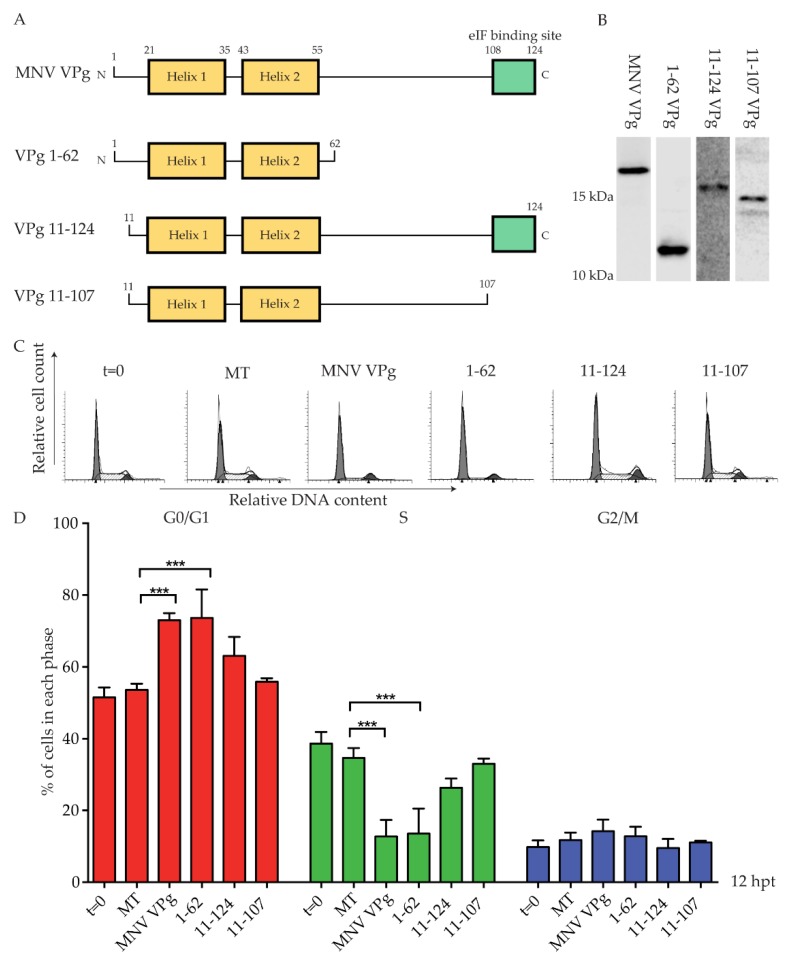
Expression of VPg 1–62 induces a G0/G1 phase cell cycle arrest. RAW-Blue cells were transfected with 4–5 µg of in vitro transcript RNA corresponding to MNV VPg, VPg 1–62, VPg 11–124, or VPg 11–107. Mock transfected (MT) cells were seeded at the time of transfection as a negative control. At 12 hpt, cells were harvested for western blot and FACS analysis of the cell cycle. (**A**) Schematic of full-length MNV VPg and synthetic constructs. Numbers indicate the amino acid position within key structural elements. (**B**) Representative western blot of full length MNV VPg and VPg truncations. Histograms (**C**) from FACS analysis were analysed on MODfit LT 3.0, and the percentage of cells in each phase of the cell cycle graphed (**D**). The results present the mean and SD from three independent experiments. Statistical significance was determined for comparisons between transfected cells and mock transfected cells using a one-way ANOVA with Dunnett’s post-test. *** *p* ≤ 0.001.

The G0/G1 phase cell cycle arrest is independent of MNV VPg amino acids Y26 and F123, key residues for genome replication and viral protein translation [16]. We therefore sought to determine if a specific region of MNV VPg was responsible for a G0/G1 phase cell cycle arrest. The structure of MNV VPg has been solved by NMR, and contains a helical core comprised of two alpha helices with unstructured N- and C-termini [20] (Figure 3). Three constructs were designed to span the regions of MNV VPg (Figure 3A). The construct VPg 1–62 includes the N-terminal region and the alpha helices, VPg 11–124 includes all regions except the first 10 amino acids, and VPg 11–107 includes the alpha helices, but excludes the N-terminal region and the eIF4G binding site (Figure 3A). Expression of VPg 1–62, VPg 11–124, and VPg 11–107 was confirmed by western blot (Figure 3B). The antibody used was generated against full-length MNV VPg; while the antibody binds to each construct, the affinity where regions of the protein were truncated is unknown, and therefore comparative expression was not attempted (Figure 3B). The N-terminal half of MNV VPg (1–62) caused a significant G0/G1 cell cycle arrest (73%) when compared to mock transfected cells (53%) (Figure 3C). Interestingly, both VPg 11–124 and VPg 11–107 had no significant effect on the cell cycle in either the G0/G1 phase or the S phase (Figure 3C). Overall, this indicates that a region within VPg 1–62 is sufficient to induce a cell cycle arrest that is contributed to, at least in part, by the first 10 amino acids.

**Figure 4:**
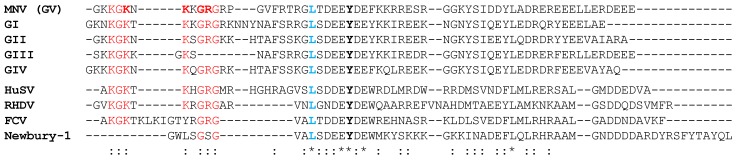
Alignment of the first 62 amino acids of VPg from different norovirus genogroups and representative members of the *Caliciviridae* family. An asterisk (*) indicates identical residues. A colon (:) indicates residues that are identical in >50% of sequences. Conserved amino acids of the N-terminal region are shown in red, and in bold red are MNV VPg residues used for point mutations. The tyrosine residue (Y) corresponding to Y26 of MNV VPg is shown in bold. A conserved leucine residue used as the junction point for the construction of chimeric VPg proteins is shown in blue. The represented genogroups are GI Norwalk virus VPg, GII Sydney 2012 VPg, GIII Jena virus VPg, and GIV Lake Macquarie VPg.

We next looked to identify conserved motifs within the first 62 amino acids of the VPg proteins tested (Figure 4). Alignment of the proteins identified a conserved N-terminal motif, KGKxKxGRG (K_3_ to G_11_ for MNV VPg), that was present in all VPg proteins confirmed to arrest the cell cycle, except for GIII (Jena virus) VPg (Figure 4), which is missing the GRG component of this motif.

**Figure 5:**
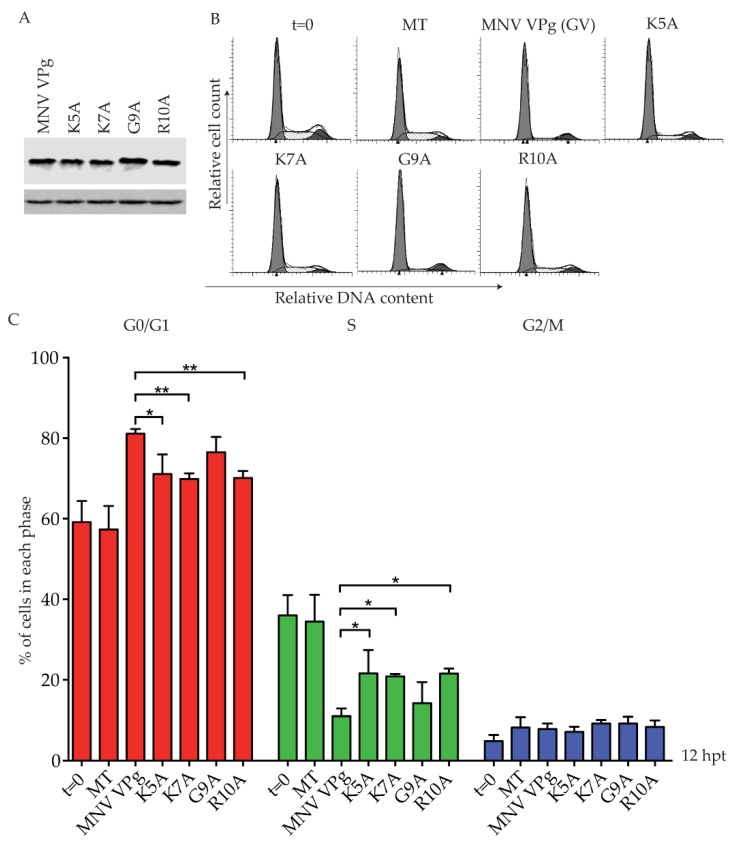
Mutation of positively charged amino acids within the N-terminal region of MNV VPg reduces the G0/G1 cell cycle arrest. RAW-Blue cells were transfected with 4–5 µg of in vitro transcript RNA, corresponding to MNV VPg or single-point mutant versions K5A, K7A, G9A, or R10A. Mock transfected (MT) cells were seeded at the time of transfection as a negative control. At 12 hpt, cells were harvested for western blot and FACS analysis of the cell cycle. (**A**) Expression of MNV VPg and point mutants was determined by western blot with actin as a loading control. (**B**) Representative FACS histograms from one of three experiments. (**C**) The histograms were analysed using MODfit LT 3.0, and the percentage of cells in each phase of the cell cycle are shown. The results present the mean and SD from three independent experiments. Statistical significance was determined for comparisons between mutanted VPg transfections and MNV VPg transfected cells using a one-way ANOVA with Dunnett’s post-test. * *p* ≤ 0.05 and ** *p* ≤ 0.01.

To investigate the role of the KGKxKxGRG motif in a G0/G1 cell cycle arrest, MNV VPg constructs were designed with single point mutations to alanine at positions K5, K7, G9, and R10. Expression of each MNV VPg mutant was confirmed by western blot (Figure 5A). Analysis of the cell cycle indicated an ~10% reduction in the G0/G1 population for K5A, K7A and R10A when compared to full-length MNV VPg, but mutation of G9 did not significantly affect the cell cycle (Figure 5C). These results indicate that lysine and arginine residues within the N-terminal region of MNV VPg are important for a cell cycle arrest.

This study has demonstrated that the N-terminal region of MNV VPg is important for a G0/G1 arrest and that alignment of VPg proteins shows a conserved KGKxKxGRG motif among the VPg proteins of the norovirus genogroups (HuSV and RHDV) (Figure 4). However, in Newbury 1 VPg the motif is almost completely absent, and in FCV VPg it is separated in two by a stretch of eight amino acids (Figure 4). Both of these proteins failed to express from in vitro RNA transcripts in RAW-Blue cells, and the cell cycle response could not be determined. We designed chimeric VPg to test the functionality of the N-terminal regions of Newbury 1 VPg and FCV VPg in the context of MNV VPg, and to address if the N-terminal region of MNV VPg is sufficient to drive a cell cycle arrest when combined with Newbury 1 virus or FCV VPg.

**Figure 6:**
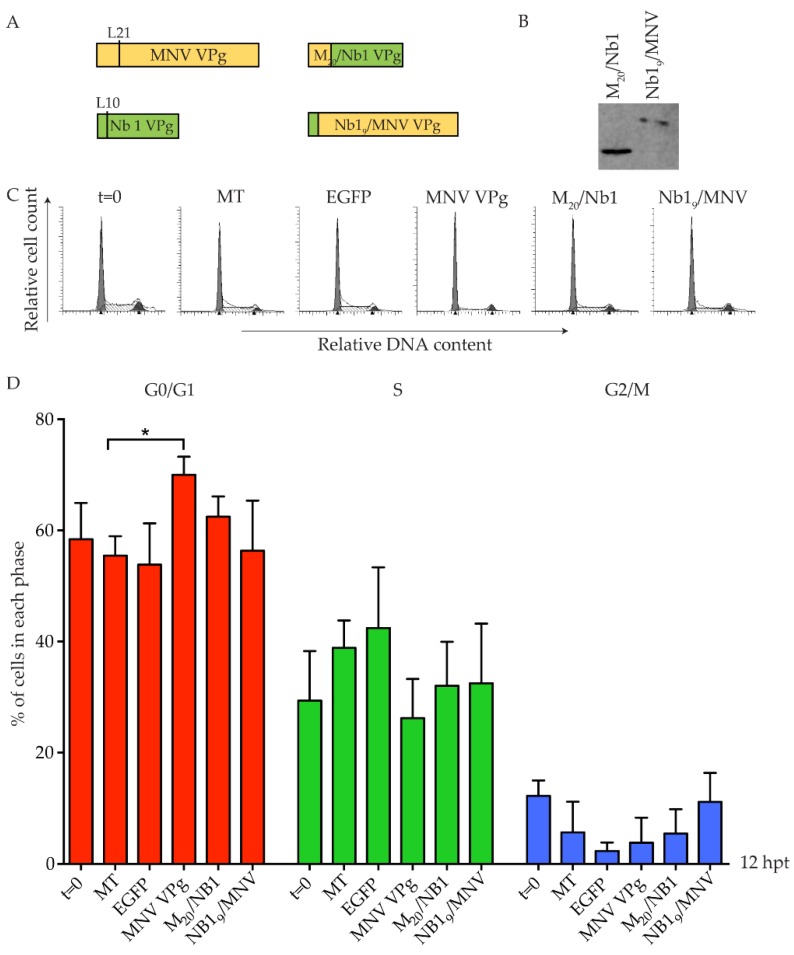
Chimeric MNV VPg and Newbury 1 VPg proteins do not induce a cell cycle arrest. RAW-Blue cells were transfected with 4–5 µg of in vitro transcript RNA corresponding to EGFP, MNV VPg, or chimeric VPg constructs. Mock transfected (MT) cells were seeded at the time of transfection as a negative control. At 12 hpt, cells were harvested for western blot and FACS analysis of the cell cycle. (**A**) Schematic for the design of chimeric VPg constructs. Chimeric constructs were designed around a conserved leucine reside of MNV VPg and Newbury 1 VPg to exchange the N-terminal region. (**B**) Expression of MNV VPg and chimeric VPg proteins was determined by purification of Strep-tag II protein on beads followed by western blot. (**C**) Representative FACS histograms from one of three experiments. (**D**) The histograms were analysed using MODfit LT 3.0 and the percentage of cells in each phase of the cell cycle are shown. The results present the mean and SD from three independent experiments. Statistical significance was determined for comparisons between transfected cells and mock transfected cells using a one-way ANOVA with Dunnett’s post-test. * *p* ≤ 0.05.

Newbury 1 virus is predicted to express a VPg product of ~65 amino acids, compared to the 124 amino acid MNV VPg [28]. Alignment of the first 62 amino acids identified a conserved leucine residue across all VPg proteins, located five amino acids immediately prior to the absolutely conserved tyrosine essential for nucleotidylation, and hence replication of caliciviruses. This leucine residue is at position 10 and 21 of Newbury 1 VPg and MNV VPg, respectively (Figure 4). Synthetic chimeric VPg constructs were designed by exchanging the N-terminal regions of Newbury 1 VPg and MNV VPg prior to the leucine residue, and a C-terminal Strep-tag II was added for protein detection (Figure 6). The resulting chimeric proteins were designated Nb1_9_/MNV VPg (representing the first nine amino acids of Newbury 1 virus fused to the MNV VPg from leucine 21) and M_20_/Nb1 VPg (representing the first 20 amino acids of MNV VPg fused to Newbury 1 virus VPg from leucine 10). Expression of each chimeric construct was confirmed by purification of Strep-tag II protein on MagStrep beads followed by western blot (Figure 6). Transfection of Nb1_9_/MNV VPg into RAW-Blue cells showed no change in the cell cycle when compared to mock transfected cells, and suggests that the N-terminal region of Newbury 1 VPg does not affect the cell cycle (Figure 6). Transfection of M_20_/Nb1 VPg also had no effect on the cell cycle, and indicates that there are region(s) of MNV VPg beyond the first 20 amino acids required to achieve a G0/G1 arrest in RAW-Blue cells, which are absent in Newbury 1 VPg.

**Figure 7:**
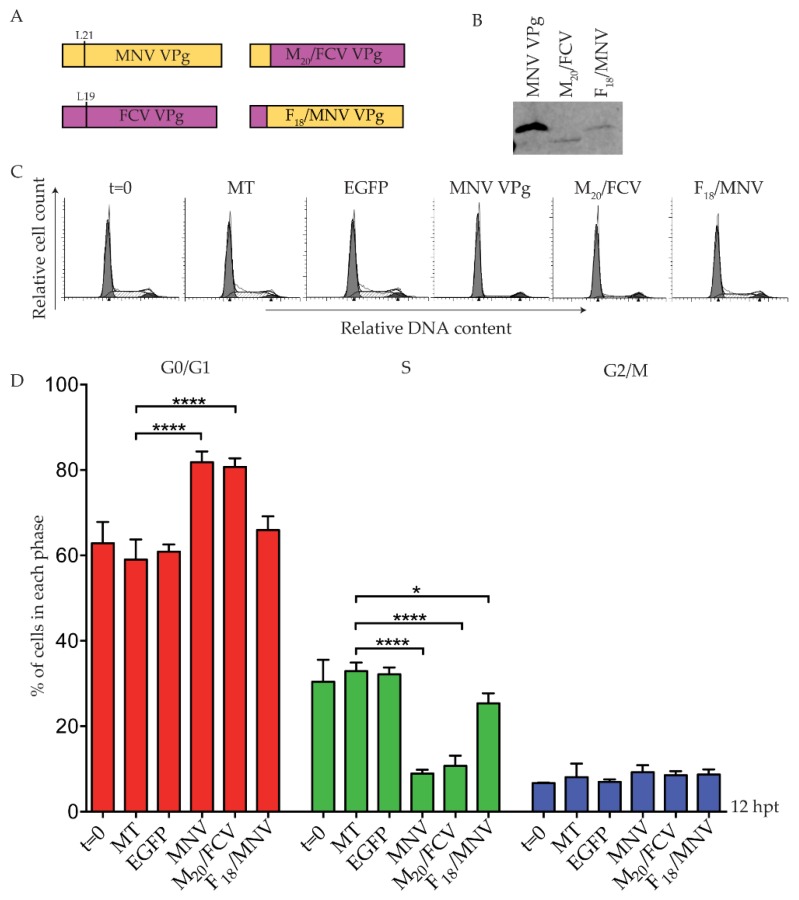
A chimeric protein M_20_/FCV VPg induces a G0/G1 arrest. RAW-Blue cells were transfected with 4–5 µg of in vitro transcript RNA corresponding to EGFP, MNV VPg, or chimeric VPg constructs. Mock transfected (MT) cells were seeded at the time of transfection as a negative control. At 12 hpt, cells were harvested for western blot and FACS analysis of the cell cycle. (**A**) Schematic for the design of chimeric VPg constructs. Chimeric constructs were designed around a conserved leucine reside of MNV VPg and FCV VPg to exchange the N-terminal region. (**B**) Expression of MNV VPg and chimeric VPg proteins was determined by purification of Strep-tag II protein on beads followed by western blot. (**C**) Representative FACS histograms from one of three experiments. (**D**) The histograms were analysed using MODfit LT 3.0, and the percentage of cells in each phase of the cell cycle are shown. The results present the mean and SD from three independent experiments. Statistical significance was determined for comparisons between transfected cells and mock transfected cells using a one-way ANOVA with Dunnett’s post-test. * *p* ≤ 0.05 and **** *p* ≤ 0.0001.

A second set of chimeric VPg constructs were designed for FCV VPg and MNV VPg, using the conserved leucine residue at position 19 of FCV VPg as the fusion point. The resulting chimeric VPg proteins were designated F_18_/MNV VPg and M_20_/FCV VPg (Figure 7). Expression of each chimera was confirmed by purification of Strep-tag II protein on beads followed by western blot (Figure 7), including expression of the predominantly FCV VPg (M_20_/FCV VPg) that could not be expressed in its unmodified form. Transfection of F_18_/MNV VPg induced a modest change in the S phase population, but this was not supported by a significant change in the G0/G1 population (Figure 7). This suggests that the N-terminal region of FCV is not functional for a G0/G1 cell cycle arrest in the system tested here. In contrast, M_20_/FCV VPg containing the N-terminus of MNV VPg fused to FCV VPg induced a cell cycle arrest comparable to MNV VPg alone (Figure 7) despite low overall protein expression (Figure 7). This implies that FCV VPg may be able to induce a cell cycle arrest in the appropriate context.

## 4. Discussion

### 4.1. Induction of a G0/G1 Arrest is a General Function of VPg Proteins from a Diverse Range of Norovirus Isolates

MNV VPg was previously reported to induce a G0/G1 phase cell cycle arrest in RAW-Blue macrophages [16]. We have established that a cell cycle arrest is a conserved function of representative VPg proteins from the other norovirus genogroups, including human noroviruses, as well as VPg proteins from the *Sapovirus* (HuSV) and *Lagovirus* (RHDV) genera. We also provide evidence that *Vesivirus* (FCV) VPg may also induce a cell cycle arrest, although that could not be confirmed. Expression of each VPg construct was confirmed by either mass spectrometry or western blot of a Strep-tag II construct. To achieve detectable levels of Strep-tag II VPg, protein lysates were purified and concentrated on MagStrep beads, and we were therefore not able to correlate the relative protein expression with the magnitude of a G0/G1 cell cycle arrest. The result for Strep-tag II GI VPg indicates that low levels of VPg expression are sufficient to induce a cell cycle arrest (Figure S1). We have interpreted a statistically significant change in the G0/G1 population as being indicative of a cell cycle arrest, regardless of protein expression level. 

Despite originating from viruses with a range of different hosts, including humans, cows and rabbits, the cell cycle arrest induced in murine macrophages by these VPg proteins is essentially indistinguishable from that of MNV VPg in the assays used in this study. The cell cycle consists of a series of sequential steps, many of which are highly conserved. Given that each of these VPg proteins induce an arrest in a single murine cell line, it seems likely that there is also a conserved mechanism.

The G1 phase of the cell cycle serves as a growth phase, during which there are high levels of ribonucleotides and high translation efficiency for genome replication and protein translation [29,30]. MNV capsid protein production and viral titre have been shown to be highest in cells that are actively progressing through the G1 phase [17]. We propose that similar to MNV, each of these respective viruses is able to induce a favorable environment, and thus gain a replication advantage within the host cell through the induction of a G0/G1 arrest.

### 4.2. A Conserved N-Terminal Motif is Required for Cell Cycle Arrest

The mechanism employed by MNV VPg to induce a G0/G1 cell cycle arrest is currently unknown. We therefore sought to identify structural elements of MNV VPg required for an arrest, which may provide insights into the mechanism. Three constructs—VPg 1–62, VPg 11–124, and VPg 11–107—were designed to span the structural and functional regions of MNV VPg, and were tested for a cell cycle arrest. The N-terminal region of MNV VPg spanning amino acids 1–62 was sufficient for a G0/G1 cell cycle arrest, while the other two constructs tested had no effect on the cell cycle. This suggests that an element(s) within the first 62 amino acids of MNV VPg is required for an arrest, and provides further evidence that the arrest is independent to the C-terminal eIF4G binding domain [16]. Further, the addition of a C-terminal tag to the VPg of GI, GII, and GV noroviruses did not affect the ability of these VPg proteins to induce a cell cycle arrest.

We performed an alignment of VPg protein sequences spanning amino acids 1–62, and identified a conserved N-terminal motif: KGKxKxGRG. The motif is conserved within all norovirus genogroups, HuSV VPg, and RHDV VPg, all of which can induce a G0/G1 cell cycle arrest. There was some variation in the degree of cell cycle arrest for different genogroup VPg proteins that contain this motif, indicating that this motif alone is not the only factor involved in cell cycle arrest (Figure 1). The ability to induce a G0/G1 arrest was associated, in part, with the positively charged amino acids K5, K7, and R10 within the N-terminal motif of MNV VPg (Figure 5).

MNV VPg mutations at K5 and K7 have previously been investigated in the context of MNV infections. Viruses expressing either of these mutations show a 10-fold reduction in virus titre compared to non-mutated viruses, suggesting that these amino acids are important for the viral lifecycle [20]. During infection, mutation of either K5 or K7 of MNV VPg does not significantly affect translation initiation factor binding or the ability of VPg to act as a primer for genome replication [20,24]. Altogether, this suggests that the reduced G0/G1 arrest observed with our K5A and K7A MNV VPg constructs is not due to the impairment of functions related to viral translation or genome replication.

Additionally, in HuNV VPg, the N-terminal region has been shown to be involved in nucleoside triphosphate (NTP) binding [31]. While native HuNV VPg binds all four NTPs at similar levels, this activity is noticeably reduced when lysine to alanine mutations at positions 2–13 are introduced [31]. NTPs, particularly purines, have been shown to regulate control of the G1 to S phase transition and progression through the S phase [32]. Depletion of the purine and guanine triggers a G1 cell cycle arrest in human neuroblastoma cell lines [33]. We would hypothesize that, for K5A at least, the cell cycle arrest is independent of NTP binding activity, as a mutation of HuNV VPg at K2–5, which includes the amino acid corresponding to K5 of MNV VPg, does not inhibit NTP binding. The role of the K7A and R10A point mutations used in this study on NTP binding has not been examined. Future experiments should focus on whether there is a direct link between NTP binding and the MNV VPg G0/G1 cell cycle arrest, focusing specifically on the role of amino acids within the N-terminal region.

It is unclear why both Newbury 1 VPg and FCV VPg failed to express as full-length proteins in this study. Newbury 1 VPg was designed from predicted cleavage sites within the ORF1 polyprotein, and has not been extensively studied [28]. The Newbury 1 VPg produced is only 65 amino acids in length compared to other VPg proteins, which range from 111–138 amino acids, and it is possible that a functional protein is not produced. In contrast FCV VPg has been detected during infection and expressed in *E. coli*; however, it could not be detected using our system [34,35,36]. Based on the amino acid alignment, we wanted to test the N-terminal regions of Newbury 1 VPg and FCV VPg to determine if they are functional in an arrest. Chimeric VPg proteins were designed to express the N-terminal regions of Newbury 1 VPg or FCV VPg in the context of MNV VPg. A cell cycle arrest was not observed for Nb1_9_/MNV VPg or F_18_/MNV VPg, suggesting that these N-terminal regions are not functional for a cell cycle arrest as chimeric proteins, although the native proteins may function differently. The N-terminal regions of Newbury 1 VPg and FCV VPg share less similarity with the consensus KGKxKxGRG motif, and further highlight the importance of this region for a G0/G1 cell cycle arrest. In contrast chimeras, expressing the N-terminal region of MNV VPg with the C-terminus of either Newbury 1 VPg or FCV VPg, were designed to investigate if the MNV VPg N-terminal region was important and could confer cell cycle arrest capability to these proteins. While M_20_/FCV VPg induced a G0/G1 arrest, M_20_/Nb1 VPg was not able to arrest cells, suggesting that an unidentified C-terminal element, absent in Newbury 1 VPg, is important. FCV VPg is more similar to MNV VPg than Newbury 1 VPg in structure, function, and protein size [20,21,27] and this finding suggests that in the correct context, FCV VPg may be able to induce a cell cycle arrest.

This work has shown that induction of a G0/G1 cell cycle arrest is a conserved function of norovirus VPg proteins that extends to VPg proteins from other members of the *Caliciviridae* family, including HuSV VPg and RHDV VPg. Within these protein sequences, we have identified a conserved motif: KGKxKxGRG. The importance of the N-terminal motif to cell cycle arrest was shown through the use of truncation, point mutations, and chimeric MNV VPg constructs. Overall, this shows that the N-terminal region of VPg is required for a G0/G1 cell cycle arrest by noroviruses.

## Figures and Tables

**Table 1:** Synthetic viral protein genome-linked (VPg) constructs to investigate the conservation of VPg-induced cell cycle arrest.

Virus ^a^	Genus and Genogroup	Accession Number	3′ Restriction Enzyme	Construct Modification ^b^
MNV	*Norovirus* GV	DQ285629	AvaI	Strep-tag II
Norwalk virus	*Norovirus* GI	AAC64602	BamHI	Strep-tag II
HuNV	*Norovirus* GII	JX459908	HindIII	Strep-tag II
Jena virus	*Norovirus* GIII	CAA90480	BamHI	No
Lake Macquarie virus	*Norovirus* GIV	AFJ21375	BamHI	No
HuSV	*Sapovirus*	X86560	HindIII	No
RHDV	*Lagovirus*	U54983	HindIII	No
FCV	*Vesivirus*	M86379	HindIII	Strep-tag II
Newbury 1 virus	*Nebovirus*	DQ013304	HindIII	Strep-tag II

^a^ MNV: murine norovirus; HuNV: human norovirus; HuSV: human sapovirus; RHDV: rabbit hemorrhagic disease virus; FCV: feline calicivirus. ^b^ Where indicated a second construct was designed to incorporate a C-terminal Strep-tag II for VPg detection. The Strep-tag II amino acid sequence was AWSHPQFEK.

## Supplementary Materials

The following are available online at https://www.mdpi.com/1999-4915/11/3/217/s1; Table S1: Detection of norovirus genogroup VPg and calicivirus VPg by mass spectrometry; Figure S1: Detection of norovirus genogroup and calicivirus VPg proteins. VPg proteins that were not detected by mass spectrometry were tagged with a C-terminal Strep-tag II and RAW-Blue cells transfected with 4–5 μg of the respective RNA. At 12 hpt cells were harvested, lysed and the protein concentrated on MagStrep “type 3” XT beads. Purified lysates were then probed for the presence of a Strep-tag by western blotting.
