# Supplementary material for: Cell Cycle Arrest is a Conserved Function of Norovirus VPg Proteins
Source: Viruses. 2019 Mar 4;11(3):217. doi: 10.3390/v11030217 (PMC6466040; doi:10.3390/v11030217)
Supplement: Supplementary file 1 [file viruses-11-00217-s001.zip › Figure S1.pdf]

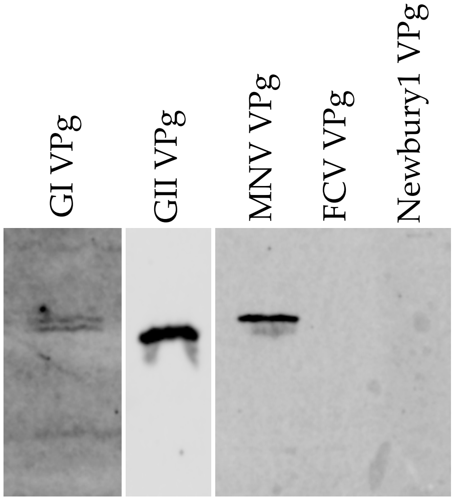

Figure S1: Detection of Norovirus genogroup and Calicivirus VPg protein. VPg proteins that were not detected by mass spectrometry were tagged with a C-terminal Strep-tag II and RAW-Blue cells transfected with 4-5 µg of the respective RNA. At 12 hpt cells were harvested, lysed and the protein concentrated on MagStrep “type 3” XT beads. Lysates were probed for the presence of a strep tag by western blotting.
