# Supplementary material for: Cell Cycle Arrest is a Conserved Function of Norovirus VPg Proteins
Source: Viruses. 2019 Mar 4;11(3):217. doi: 10.3390/v11030217 (PMC6466040; doi:10.3390/v11030217)
Supplement: Supplementary file 1 [file viruses-11-00217-s001.zip › Table S1.pdf]

Table S1: Detection of norovirus genogroup VPg and calicivirus VPg by mass spectrometry

Viral VPg	VPg Sequence[a]
MNV (GV) VPg	GKKGKNKKGRGRPGVFRTRGLTDEEYDEFKKRRESRGGKYSIDDYLADREREEELLERDEEEAIFGDGFGLKATRRSRKAERAKLGLVSGGDIRARKPIDWNVVGPSWADDDRQVDYGEKINFE
GIII VPg	SKKGKKGKSNAFSRRGLSDEEYDEYKKIREDRGGNYSIQEYLEDRERFERDLAERQADDADYDVGAIRQKYFGRGKAAKAQRKRIDWNPTGPLWADDDRQVDYDEVIDFQ
GIV VPg	GKKKGKNKQGRGRKHTAFSSKGLSDEEYEEFKQLREEKGGKYSIQEYLEDRDRFEEEVAYAQACGGDCDDIEISRIRNSIFRPSRKQRKEERVKLGLVTGSEIRKRKPDDFQPKGKLWADDERTVDYNEKLDFE
RHDV VPg	GVKGKTKRGRGARVNLGNDEYDEWQAARREFVNAHDMTAEEYLAMKNKAAMGSDDQDSVMFRSWWTRRQLRPDEDQVTIVGRGGVRNEVIRTRARQAPKGPKTLDDGGFYDNDYE
HuSV VPg	AKGKTKHGRGMRHGHRAGVSLSDDEYDEWRDLMRDWRRDMSVNDFLMLRERSALGMDDEDVARYRAWLEIRAMRMAGGAYTHATIIGRGGVRDEIIRTSPRRAPTRPQQHYEEE

[a] Full VPg sequence for each protein with amino acids detected by mass spectrometry shown in red.
